# Applications of mesenchymal stem cell technology in bovine species

**DOI:** 10.1186/s13287-019-1145-9

**Published:** 2019-01-24

**Authors:** Amanda Baracho Trindade Hill, Fabiana Fernandes Bressan, Bruce D. Murphy, Joaquim Mansano Garcia

**Affiliations:** 1 Department of Preventive Veterinary Medicine and Animal Reproduction, São Paulo State University, Via de Acesso Professor Paulo Donato Castelane - Vila Industrial, s/n, Jaboticabal, SP 14884-900 Brazil; 2 Centre de Recherche en Reproduction et Fertilité, Faculté de Médecine Vétérinaire, Université de Montréal, Saint Hyacinthe, QC J2S 7C6 Canada; 3 Campus Fernando Costa, University of São Paulo, Av. Duque de Caxias Norte, 225 - Zona Rural, Pirassununga, SP 13635-900 Brazil

**Keywords:** Mesenchymal stem cells, Cell culture, Pluripotent, Livestock, Cow, Cattle, Biotechnology, Cellular therapy, Regenerative medicine, Translational research

## Abstract

Mesenchymal stem cells (MSCs) have received a great deal of attention over the past 20 years mainly because of the results that showed regeneration potential and plasticity that were much stronger than expected in prior decades. Recent findings in this field have contributed to progress in the establishment of cell differentiation methods, which have made stem cell therapy more clinically attractive. In addition, MSCs are easy to isolate and have anti-inflammatory and angiogenic capabilities. The use of stem cell therapy is currently supported by scientific literature in the treatment of several animal health conditions. MSC may be administered for autologous or allogenic therapy following either a fresh isolation or a thawing of a previously frozen culture. Despite the fact that MSCs have been widely used for the treatment of companion and sport animals, little is known about their clinical and biotechnological potential in the economically relevant livestock industry. This review focuses on describing the key characteristics of potential applications of MSC therapy in livestock production and explores the themes such as the concept, culture, and characterization of mesenchymal stem cells; bovine mesenchymal stem cell isolation; applications and perspectives on commercial interests and farm relevance of MSC in bovine species; and applications in translational research.

## Background

Stem cell biology has been a very active field over the past decade. The number of studies has increased significantly, and this has been accompanied by breakthroughs in several areas in the field. Stem cell therapy has rapidly advanced prospects for personalization of therapy, tissue engineering, and chronic and regenerative disease mitigation. In human and veterinary research, stem cells derived from adult tissues are promising candidates for disease treatment, specifically for their plasticity, their low immunogenicity, and their high anti-inflammatory potential [1]. In addition, mesenchymal stem cells (MSCs) characteristically produce bioactive mediators and adhesion molecules that help to inhibit scar formation and apoptosis, increase angiogenesis, and stimulate intrinsic progenitor cells to regenerate their functionality [2, 3]. Stem cell therapy offers potential solutions for a variety of chronic diseases for which current pharmacologic therapy does not provide effective treatment [4] as well as for some surgical procedures. In addition, an exciting new step in cellular therapy is the use of MSC for immune modulation [5].

Veterinary regenerative medicine research has focused principally on companion and sport animals, but a critical reading of published findings, combined with select papers published in livestock species, allows us to generate valuable insights into the future of regenerative medicine applications in animal husbandry. Among all domesticated species, cows have crucial importance in the economics of the livestock industry, with 69.6 million tons of meat and 811 million tons of milk produced worldwide in 2017 [6, 7]. There are several medical conditions, such as mastitis, lameness, and fracture that can reflect negatively on meat and milk production as well as on reproductive efficiency in cattle. For cattle with high economic or genetic potential, these losses pose significant costs to the owner, who is therefore willing to employ expensive and effective treatments [8]. In this review, we discuss the importance of stem cell technology in bovine species in order to address disease and injury with both animal welfare and economic benefits.

## The nature of mesenchymal stem cells

The term “stem cell” emerged in the nineteenth century, describing mitotically quiescent primordial germ cells capable of regeneration of a variety of tissues [9]. Stem cells are defined by their ability to self-renew and by their potential to differentiate into functional cells under appropriate conditions [10]. In animals, two classes of stem cells have been identified: embryonic stem cells (ESC) and adult (somatic) stem cells (ASC) [11], which include mesenchymal stem cells, hematopoietic stem cells, and tissue-specific stem/progenitor cells [12].

MSCs are responsible for tissue turnover; therefore, when tissue repair is necessary, these cells can be stimulated to proliferate and differentiate, resulting in their presence in many [10], if not all [2], tissues. In addition, MSCs display important features that render them valuable for cell therapy and tissue engineering such as their low immunogenicity, high anti-inflammatory potential [1], ability to modulate innate immune responses [5], bioactive mediation and adhesion capacity to inhibit scar formation and apoptosis, increased angiogenesis, and stimulation of intrinsic progenitor cells to regenerate their functionality [3]. Due to their clinically relevant characteristics, MSCs have received more attention than the other ASC types.

During early embryogenesis, the trophectoderm differentiates into extraembryonic tissues, while the inner cell mass of the embryo, populated by embryonic stem cells, gives rise to the embryo itself, thus being able to differentiate into all cell types that form the body [11]. In contrast, it was a generally held belief that MSCs have restricted differentiation ability, being able to differentiate into mesenchymal lineages only. In the early 2000s, some discussion took place regarding the veracity of the definition of mesenchymal stem cells, concerning their potential to differentiate into non-mesenchymal lineages and whether the differences that seemed to exist between ESC and MSC had narrowed to a point that it was questionable whether they existed at all [13]. In 2002, it was shown that bone marrow-derived cells expressed some pluripotent markers, such as Oct-4, Rex-1, and SSEA; were able to differentiate into three germ layers in vitro; and when injected into an early blastocyst, were able to contribute to all organs [14]. The number of studies investigating the pluripotent ability of MSC has grown recently, and many researchers have reported cells derived from bone marrow [15, 16], adipose tissue [17], ovarian tissue [18, 19], placenta [20], and uterus [21] that express pluripotent markers. MSCs derived from several different species, including bovine, have been shown to differentiate into mesodermal, endodermal, and ectodermal lineages [16, 22]. A relevant clinical difference between ESC and MSC is that MSCs do not form teratomas when injected in vivo [14, 17], which is favorable for their clinical use.

Rigorous evaluation of the differentiation capacity of MSC is a critical step in the solidification of support for their redefinition as pluripotent. In order to study the functionality of MSC, experiments were performed to evaluate the transdifferentiation of MSC in vivo. Studies have shown the ability of MSC to transdifferentiate into various types of skin cells, islet-like cell clusters, and renal epithelium cells [23–25]. These three studies are just a few examples of the considerable amount of data that has been collected over the past decade supporting the transdifferentiation potential of MSC when transplanted in vivo. Considering these results together, MSCs have been proven to functionally differentiate into three germ layers. If MSCs express pluripotent markers and have the ability to differentiate in vitro into three germ layers and transdifferentiate in vivo into three germ layers, perhaps there is a lack of precision concerning terminology in some papers when they are called multipotent.

## Mesenchymal stem cell culture

Cell culture begins after mechanical or enzymatic disaggregation of the original tissue and can be performed under various conditions such as in an adhesive layer, a solid substrate, or in a suspension culture. It is well established that MSCs adhere to plastic substrate culture plates [26], a characteristic condition of MSC that arises after tissue disaggregation. Disaggregation is achieved by proteolytic enzyme digestion that is very effective at isolating cells from a tissue; however, it also has the potential to damage them. According to Gazit [10], MSC derived from adipose tissue can be easily isolated after enzymatic treatment with collagenase. This enzyme is the most frequently used for isolation of MSC due to its ability to cleave collagen connections [27]. The optimum concentration of the enzyme, the incubation time, and the temperature must be carefully monitored during isolation [28].

Different protocols have been used to isolate, expand, and characterize MSC. One common protocol, based on cell adherence to the plastic during the first 48–72 h of culture, is effective, though typically results in a heterogeneous population of cells [19, 29, 30]. To select a homogenous or a desirable population of MSC, more stringent isolation protocols have been proposed. These include the use of different cell culture media [31], cell sorting [15, 32, 33], and cell adherence to the plastic during the first 3 h of culture [18, 19].

MSCs have the capacity to expand several times in culture, maintaining their growth potential and plasticity, with a doubling time which is variable according to the tissue and initial plating density [34]. Each time that the cells fill the flask culture area, they need to be enzymatically removed from the flask for further re-cultivation, a process defined as cell passage [35].

In order for the cells to become able to survive, proliferate, and differentiate in vitro, the culture system must emulate the in vivo conditions of the cells’ original tissue [36]. The cells must be maintained in an incubator with 5% CO_2_, which facilitates pH maintenance in the culture medium [37], at the physiological temperature optimal for the donor species.

Supplementation of the medium should be performed to mimic in vivo conditions in order to sustain cell growth. Fetal bovine serum (FBS) is used in the cell culture medium as a source of growth factors and a vital nutrient, which supports expansion and attachment of MSC to the culture plate [38]. The use of antibiotics is important to prevent contamination, and it is necessary to evaluate the type of contamination that cells may be exposed to and potential toxicity of the dose when choosing which antibiotic to use. The most commonly used antibiotics are penicillin and streptomycin, making an effective and relatively non-toxic combination at the concentrations of 100 U/mL and 100 mg/mL, respectively [28].

## Mesenchymal stem cell characterization

Stem cells are defined by their ability to self-renew and by their potential to differentiate into functional cells under the right conditions [10]. Different protocols have previously been reported regarding the isolation, characterization, and expansion of MSCs. Generally, MSCs express CD105, CD73, and CD90 and lack the expression of hematopoietic markers such as CD45, CD34, CD14 or CD11b, CD79α or CD19, and HLA-DR surface molecules [26]. However, MSCs from different species do not express all the same markers [39]. Additionally, it has been demonstrated that MSCs isolated from different tissues express different markers and have different plasticity [40]. A summary of MSC surface markers in different species can be found in Table 1**.**

**Table 1:** Cell surface markers in different species

Cell surface markers		Species	References
Positive	Negative		
CD105, CD73, CD90	CD45, CD34, CD14, CD19, HLA-DR	Human	[26]
STRO-1, CD44, CD90, CD105	CD73, CD45, CD34	Canine	[18, 41]
CD105, CD90, CD44	CD34, MHC II	Equine	[42]
CD29, CD166, CD105, CD73, CD44, CD90	CD45, CD34	Bovine	[1, 16, 22, 45–48]

Canine MSCs, for example, have been shown to be positive for the markers STRO-1 and CD44 and negative for CD73, a classic human MSC marker [41]. Later, when MSC molecular markers from canine adipose tissue and ovarian tissue were compared, it was found that both derived cell types expressed CD44, CD90, and CD105; however, ovarian MSC-derived cells expressed higher levels of OCT4 than adipose-derived cells [18].

In equine species, MSCs from bone marrow (BM-MSC), adipose tissue (AT-MSC), and umbilical cord (UC-MSC) were compared with respect to their immunophenotypic characterization and differentiation potential. It was shown that all three sources of MSC expressed CD105, CD90, and CD44; however, UC-MSC had lower expression of CD90 than the other sources. Interestingly, BM-MSC and AT-MSCs showed faster in vitro differentiation than UC-MSC [42].

In humans, BM-MSC, AT-MSC, and UC-MSC were compared and demonstrated to express varying levels of certain MSC markers, including lower expression of CD90 and higher expression of CD105 by UC-MSC than the other sources [43], similar to the results found in equine species. Human adipose tissue-, bone marrow-, umbilical cord blood-, and nasal septum (NSP-MSC)-derived cells were compared with regard to their pluripotency markers. It was shown that AT-MSC had the highest expression of Sox2, Klf4, and Lin28 but the lowest of Oct4 and cMyc genes. Meanwhile, BM-MSC had more expression of Nanog and cMyc and the lowest expression of Rex1. UC-MSC and NSP-MSC had more expression of Rex-1 and Oct4, respectively [44].

**Fig. 1:**
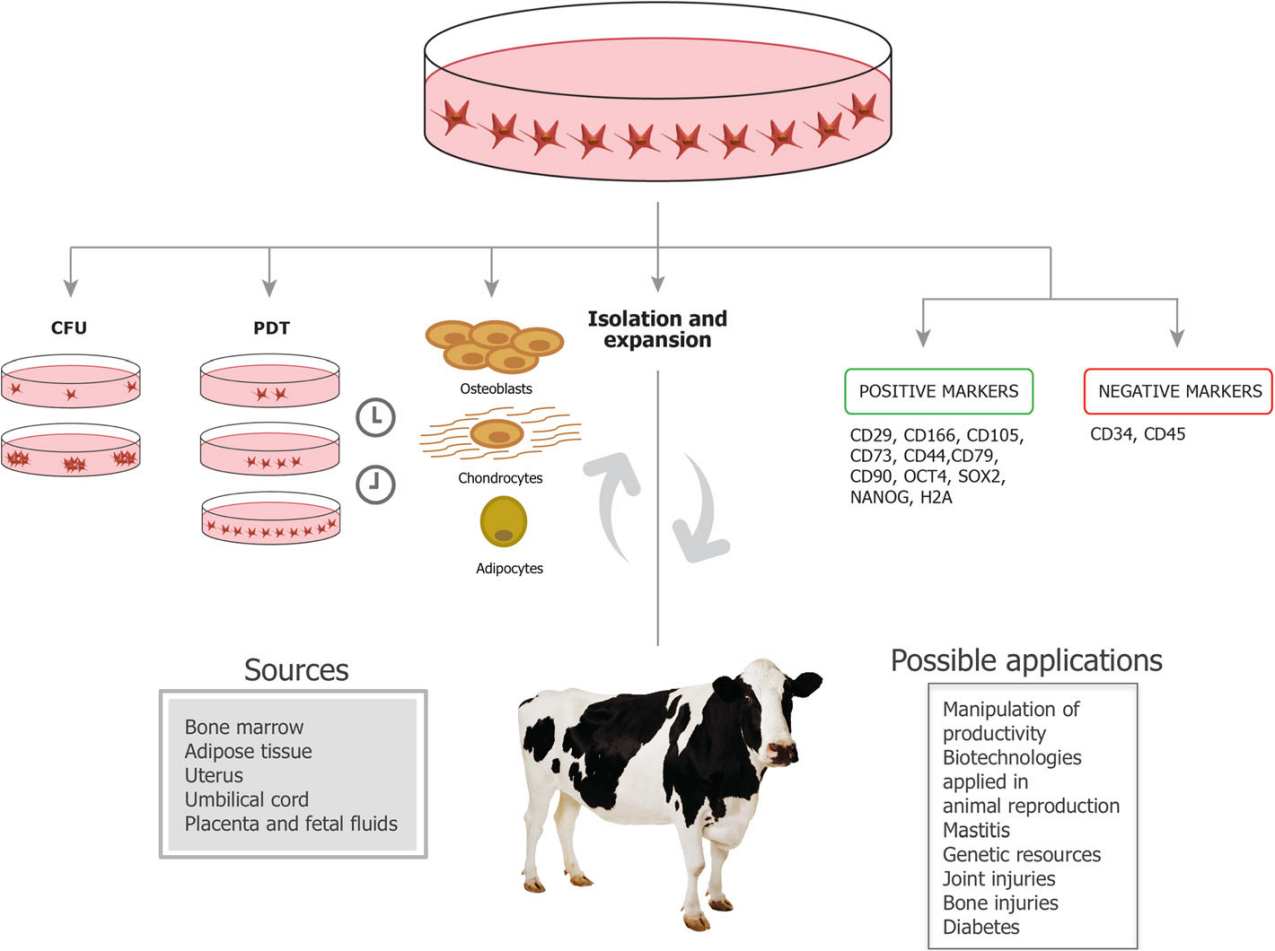
Isolation, characterization, and potential applications of bovine mesenchymal stem cells. Bovine mesenchymal stem cells have been isolated from the uterus, umbilical cord, bone marrow, adipose tissue, placenta, and fetal fluids. After isolation, the cells are expanded and characterized to prove their mesenchymal nature. The ability to self-renew is an important feature to be characterized in vitro and can be done by analysis of colony unit formation (CFU) and population doubling time (PDT). The cells need to show the ability to differentiate into osteogenic, chondrogenic, and adipogenic lineages. The bovine-isolated cells have already been shown to be positive for some mesenchymal and pluripotent markers and negative for hematopoietic markers. After characterization, the cells can be injected into the animal for therapeutic applications. Uses of bovine MSC for treatment of joint injuries, mastitis, and bone injuries; preservation of genetic resources; manipulation of productivity; and use in biotechnology applied in animal reproduction have all been suggested

Regarding bovine species, some characterization has taken place, as shown in Fig. 1. Bovine MSCs derived from different tissues have been shown to be positive for mesenchymal markers related to adhesion such as CD29, CD166, CD105, surface enzymes such as CD73, receptors such as CD44, and glycoproteins such as CD90 [1, 16, 22, 45–48]. Interestingly, bovine MSC also expressed pluripotency markers such as OCT4, SOX2, and NANOG [1, 16, 21, 45, 46], supporting the idea that MSCs have the potential to be pluripotent and differentiate into three germ layers, which was previously shown by the successful differentiation of bovine MSC into osteoblasts, lipoblasts, hepatocytes, islet cells, and neurocytes [22].

Regardless of the cell source or isolation procedure, MSC should express CD105, CD73, and CD90 and lack the expression of hematopoietic markers such as CD45, CD34, CD14 or CD11b, CD79α or CD19, and HLA-DR surface molecules, as established by the International Society for Cellular Therapy as the minimum criteria for MSC characterization in humans [26]. These are used as de facto criteria in other species as well. Currently, there are no specific criteria for mesenchymal stem cell characterization in cattle. Future challenges include defining a standard characterization protocol of MSC in this species. Despite the lack of commercial antibody availability for cattle, PCR can be used for the study of MSC molecular profile. For translational medicine, a complete evaluation of different sources of MSC needs to be performed, in order to evaluate similarities between human and bovine MSC.

## Sources of bovine mesenchymal cells

### Bone marrow

Bone marrow was the first tissue described as a source of plastic-adherent, fibroblast-like cells that develop fibroblastic colony-forming units (CFU-F) when seeded on tissue culture plates. MSCs derived from bone marrow were first isolated and identified in mice and were described as non-hematopoietic cells with the potential to differentiate into mesodermal tissues, such as adipocytes, osteoblasts, chondrocytes, and skeletal muscle cells [49].

In cattle, bone marrow has been the source for MSC in several studies [16, 46, 50–52]. In this procedure, marrow cells are aspirated from calves and isolated for further analysis. Many reports with bovine BM-MSC focused on chondrogenic differentiation [50–52]. Spontaneous chondrogenesis of bovine MSC in pellet culture occurred without the addition of any external bioactive stimulators, i.e., factors from the transforming growth factor (TGF)-β family, previously considered necessary [50]. The same group isolated bovine MSC from eight calves and induced them to undergo osteogenic, chondrogenic, and adipogenic differentiation [51]. One year later, the same group analyzed the MSC chondrogenic response during culture on different types of extracellular matrices (ECM). Bovine MSCs were cultured in monolayer as well as in alginate and collagen type I and II hydrogels, in both serum-free medium and medium supplemented with TGF-β1. Differentiation was most prominent in cells cultured in collagen type II hydrogel, and it increased in a time-dependent manner. TGF-β1 treatment in the presence of collagen type II provided more favorable conditions for the expression of the chondrogenic phenotype. It was concluded that collagen type II has the potential to induce and maintain MSC chondrogenesis, but in the presence of TGF-β1, the cells expressed higher transcript levels of genes associated with differentiation, suggesting a higher fidelity differentiation [52]. The presence of BM-derived MSC with a pluripotent profile was demonstrated in later experiments. The cells were adherent to plastic surfaces and exhibited fibroblast-like morphology. In addition, the cells expressed pluripotent markers, such as OCT4, SOX2, and NANOG, as well as typical MSC markers, including CD29, CD90, and CD105. When the cells were isolated from fetal BM, they exhibited fibroblast-like morphology and were able to differentiate into hepatogenic and neurogenic lineages. The cells were not only positive for MSC markers CD29 and CD73 but also for the pluripotency markers, whereas they were negative for hematopoietic markers CD34 and CD45 [16].

### Adipose tissue

Currently, bone marrow and adipose tissue are the main sources of MSC in veterinary medicine [53]. However, AT-MSCs have some advantages over BM-MSC, including faster development in vitro [53], easier isolation, and higher density stromal cells [54]. To date, there are only two studies in bovine species with MSC isolated from adipose tissue [47, 55]. In both studies, cells exhibited fibroblast-like morphology and were able to differentiate into osteogenic, chondrogenic, and adipogenic lineages; they expressed different MSC markers in each of the studies. In one study, cells were positive for CD105, CD73, CD29, CD90, and H2A markers and negative for CD45, CD34, and CD44 markers [55], while in the other study, cells were positive for CD90, CD105, and CD79 and the negative for CD45, CD34, and CD73 [47]. MSCs are known to demonstrate considerable variability between populations in their proliferation, differentiation, and molecular phenotype [39, 40, 56].

### Umbilical cord

The umbilical cord has two sources of MSC. One is the cord blood, from which the cells are isolated by density gradient, and the other is the cord tissue, from which the cells can be disaggregated by enzymatic action. The cord blood is collected non-invasively and represents an alternative source of stem cells when compared to adipose tissue and bone marrow. In addition, the high availability and lower immunogenicity of umbilical cord blood cells compared to other sources of stem cells such as bone marrow have made them a viable and valuable source for cell therapy [45].

It was reported that cells isolated from the umbilical cord blood of humans have more MSC volume and greater plasticity, are genetically more flexible than bone marrow MSC, and also, as noted above, produce a less prominent immune response [57, 58]. While MSCs derived from umbilical cords in human, murine, and avian species have been the subject of many investigations, little is known about these cells in livestock species [22]. The first study that isolated bovine MSC from the umbilical cord blood observed that the cells grew into monolayer cell sheets and could be expanded into high passages. In addition, the cells expressed OCT4 and CD73 and were able to differentiate into osteogenic, chondrogenic, and adipogenic lineages [45]. In another study, isolated cells were sub-cultured to passage 32 and expressed CD29, CD44, CD73, CD90, and CD166 [22]. Moreover, those cells were able to differentiate into osteoblasts, lipoblasts, hepatocytes, islet cells, and neurocytes, indicating their potential use for experimental and clinical applications for bovine, and very importantly showing evidence that MSCs have the potential to differentiate into non-mesodermal lineages [22].

### Placenta and fetal fluids

The placenta performs a number of very important roles during pregnancy, including being responsible for the supply of nutrients, production of hormones, elimination of waste, and facilitation of gas exchange [59]. The placenta can be isolated easily by non-invasive harvest after delivery without any ethical or moral concern [20]. Only one study with bovine placenta-derived mesenchymal stem cells has been published, in which the authors successfully differentiated islet-like cells from the placental stem cells. The isolated cells expressed typical mesenchymal stem cell markers, including CD73 and CD166, and a pluripotent marker, OCT4, but not hematopoietic markers, such as CD45 [20].

Regarding fetal fluids, it has been reported that the amnion and amniotic fluid are abundant sources of mesenchymal stem cells that can be harvested at low cost and without ethical conflict [1]. The authors isolated MSC from amniotic fluid, and the cells exhibited fibroblast-like morphology only starting from the fourth passage, being heterogeneous during the primary culture. Immunofluorescence results showed that amniotic fluid MSCs were positive for CD44, CD73, and CD166 but negative for CD34 and CD45. In addition, the cells expressed OCT4 and, when appropriately induced, were able to differentiate into ectodermal and mesodermal lineages [1].

### Uterus

The endometrial stromal cells are dynamic, growing, and differentiating throughout the estrous cycle and pregnancy [60]. In addition, these cells are known to modulate the immune system and could have clinical applications for human and animal health [48]. Some studies have isolated and characterized bovine mesenchymal stem cells in the endometrium [21, 48, 60]. The cells had fibroblast-like morphology, and when cultured in a specific osteogenic medium, they rapidly developed the characteristics of mineralized bone [60]. The endometrium-derived cells were found to express MSC markers such as CD29 and CD44 [31] and pluripotent markers such as OCT4, SOX2, and c-KIT [21]. Moreover, the cells demonstrated excellent clonicity, differentiation potential in mesodermal lineages, and excellent maintenance of quality after the cryopreservation process [48]. A recent report showed the ability of endometrial cells to adhere to the plastic culture dishes, displaying fibroblast-like morphology, high proliferative capacity, and the ability to differentiate into chondrogenic, osteogenic, and adipogenic lineages.

## Therapeutic delivery of mesenchymal stem cells

To achieve the best response after cell therapy, the general health of the patient, time of cell application, cell type, delivery route, and number of applications must be considered [35]. Following stem cell derivation, cell expansion is needed for subsequent transplantation into the patient [61]. In addition, cryopreservation of these cells can provide a ready source of abundant autologous stem cells [62]. Cryopreservation of bovine MSC may be achieved successfully with no change in the characteristics between fresh and thawed cells [48]. The delivery of the cell preparation should take place rapidly in order to avoid changes in cell viability and to prevent biological contamination of the cells [61]. Moreover, it has been suggested that early administration of stem cells is presumed to be more advantageous than attempting treatment when fibrous scar tissue has already been formed [63].

The most effective delivery method depends on the condition that is being treated. Intravenous administration is possible due to the ability of MSC to migrate across the endothelium and home to injured tissues [10, 39]. However, cells can become trapped in the lungs [39]. Thus, direct injection to the injured tissue provides a more convenient method [64], aiming a high concentration of MSC at the injury site without the risk of cell migration to other sites in the body [10]. In cases in which relevant structural defects are present, such as segmental bone, articular cartilage, and soft tissue defects, the cells need to be delivered by a carrier in order to have a substrate to control cell adhesion as well as the location of the cells in vivo, and to form a template for the formation of new tissue [64]. Recently, decellularized tissue has proven to be a promising option for scaffold construction [65]. The bovine model in particular has an advantage when compared to smaller animal models such as mice, due to the larger quantity of tissue to be decellularized, providing a much closer analogy to human conditions for eventual translational applications in organ construction and tissue engineering [66].

## Bovine mesenchymal stem cell therapy

### Mastitis

The dairy industry is a multi-billion dollar industry, with 811 million tons of milk produced in 2017 [7]. Clinical mastitis significantly reduces milk production and animal value. It has a severe impact on udder tissue and is also an animal welfare issue. Very importantly, the damage caused by mastitis cannot be mediated or reversed with current therapeutic strategies. Bovine mammary stem cell therapy offers significant potential for the regeneration of the udder tissues such that they could be replaced/repaired with minimal side effects [67]. Furthermore, the anti-inflammatory properties of the MSC [1] could potentially reduce the severity of the disease.

Stem cells modified with therapeutic agents may also be employed to combat mastitis. It has been reported that cloning the bovine lactoferricin (*LFcinB*) gene into the PiggyBac transposon vector is a feasible means of creating MSCs with heterologous expression of the hybrid antibacterial peptide LfcinB [68]. These cells would then confer their high antibacterial properties against bovine mastitis origin *Staphylococcus aureus* and *Escherichia coli* directly into the mammary gland, providing strong innate udder immunity to fight against intramammary infections [68]*.* This study represents a template for cost-effective expression of other antimicrobial peptides in genetic engineering. In addition to the therapeutic advantage of this approach, because of the high milk production ability, bovine mammary glands can be used as bioreactors for the production of proteins on a large scale for the pharmaceutical industry [68].

### Biotechnology applied in animal reproduction

Nuclear transfer was successfully performed in amphibians in the 1950s and in mammals some 30 years later. Dolly the sheep was the first mammal to be cloned by somatic nuclear transfer [69]. The goal of nuclear transfer research was to introduce precise genetic modifications in livestock species by making the desirable modifications in cells used as nuclear donors [70]. MSC could be used to produce transgenic animals for the improvement of the animal’s health as well as for biomedical interest, for example, to produce cows resistant to mastitis [71] and to recover proteins, such as human α-lactalbumin, from milk [72].

Another interesting possibility that arose from the development of nuclear transfer was that of cloned human embryos produced with the purpose of further establishment of patient-specific ES cells for regenerative medicine [70]. However, bioethical issues and related regulations hampered the attempts at production of human embryonic stem cells. To overcome that issue, in 2006 [73], somatic cells were reprogrammed to a pluripotent state by introducing transcription factors (OCT3/4, SOX2, KLF4, and C-MYC) into their genome. These cells were called induced pluripotent stem cells (iPS) and had similar characteristics to ESC, including the ability to originate tissues from the three germ layers both in vitro and in vivo [73]. Despite the advantages of iPS, there are still several ethical issues related to their application, such as genetic instability, tumorigenicity, and differentiation. Also, efficient methods for cell transplantation need to be investigated further [74]. The low tumorigenicity and high differentiation potential have made MSC a very promising source of cells for the treatment of degenerative and inherited diseases [14].

Nuclear transfer technique is based on the transfer of the nucleus from a donor cell into an oocyte or early embryo from which the chromosomes have been removed [70]. The most important drawback of this technique is the inability of the ooplasm to eliminate epigenetic markers and restore the genetic material of the donor nucleus to the embryonic totipotent state [75]. Many studies have focused on resolving this inability, due to the importance of chromatin structure in the cell reprogramming process [76]. One of the areas that have been explored by these studies is the use of mesenchymal stem cells for somatic nuclear transfer, which has been suggested in bovine species [47, 55, 76]. For example, it was shown that the epigenetic status of bovine adipose-derived MSC was variable during culture. Of the cell passages examined in this study, passage 5 seemed to be the most efficient in the performance of nuclear transfer due to its high level of stemness, multipotency, and the low level of chromatin compaction [76]. The embryo production rate was also shown to improve when embryos were co-cultured with MSC [77], representing in yet another way the importance of MSC in addressing commercial goals.

### Bone injuries

Although some bone fractures and small defects can regenerate, there are conditions in which tissue loss is too extensive, as well as cases of non-union fractures and other critical-size defects where osteogenesis does not physiologically occur [10]. This represents another opportunity in which the application of MSC could upregulate the body’s regenerative process to improve patient recuperation.

The events associated with bone healing have been chronicled reviewed [78]. When a bone fracture occurs, the inflammatory response increases the blood supply to the region. Cellular recruitment initially leads to the replacement of the fracture hematoma with fibrous tissues and, progressively, cartilaginous matrix, which is subsequently replaced by bone through endochondral ossification in both the periosteal and endosteal callus. MSCs reside in the bone marrow in low densities, and the recruitment of MSC to the fracture site is critical. This recruitment occurs by way of a chemotactic stimulus and results in the homing of circulating stem cells to the site of injury. Once these cells arrive, they begin participating in repair mechanisms [78].

The reconstruction of large bone segments is a relevant clinical problem. Preclinical and clinical data are accumulating to support the use of MSC to enhance bone repair and regeneration [79]. There are no clinical data on the use of mesenchymal stem cells for bone repair in cattle, although the ability of MSC to differentiate into the osteogenic lineage has been shown [1, 21, 22, 46, 51, 56, 60, 80–83].

Attitudes in the livestock industry have shifted towards the preservation of the commercial viability of individual animals with high genetic value, leading in turn to an increase in medical expenditure to keep those animals healthy. Owners are frequently willing to elect expensive treatments, even when the prognosis is poor, when cattle have high economic or genetic potential [8]. This notwithstanding, a number of criteria should be carefully analyzed when deciding the best treatment for a bone fracture, such as cost and success rates of the treatment, the value of the animal, and the location and type of fracture. Unlike horses, only rodeo livestock cattle need to perform athletically; thus, musculoskeletal integrity is less of an issue. However, fractures can result in a loss of meat and milk production and interfere with reproductive efficiency, including nefarious effects on natural breeding and impairment of embryo and semen production as well [8]. Thus, MSC could represent an important auxiliary source in the treatment of bone fracture for cattle for multiple reasons, including their anti-inflammatory potential [1], their ability to increase angiogenesis, and their ability to stimulate intrinsic progenitor cells to regenerate tissue functionality [3]. MSC treatment has the potential to reduce animal recovery time and reduce economic loss associated with bone injury, reducing the time for repair that can negatively influence milk and meat production and interfere with natural breeding, as mentioned above. In addition, the reduction of the recovery period can improve the outcomes for cattle with aggressive behavior, in which conventional treatment would be impractical due to the necessary motion constraints and temperament issues.

### Joint injuries

In cattle, chronic osteoarthritis (OA) has been reported to be a significant cause of infertility in bulls [84], leading to economic loss and decrease in animal value. OA is a degenerative disease of the articular cartilage, which causes the release of pro-inflammatory cytokines [85]. The molecules involved in the OA process include growth factors, transforming growth factor β (TGF-β1), and cytokines and chemokines such as IL-8 [86, 87]. These molecules influence a wide range of biological processes that include cell proliferation, differentiation, migration, and apoptosis [88]. In horses, the efficacy of stem cells for the treatment of OA has been evaluated in the form of experimental and clinical studies, with more favorable results for bone marrow-derived cells than adipose-derived cells. The fact that MSCs secrete paracrine signaling molecules and trophic factors that influence cell response to injury and modulate the innate immune response [5] demonstrates the potential use of those cells for OA treatment in cows. In this species, there are no current clinical data, although some studies have demonstrated the isolation of MSC and their potential to differentiate into the chondrogenic lineage [21, 45, 50–52, 55, 81, 83, 89–91]. Methods are evolving to achieve this goal. To induce MSC to undergo chondrogenic differentiation, factors that support strong cell-cell interaction, growth factors, and an environment which maintains spherical morphology such as polymer gels have been shown to be required [52]. It has further been reported that the age of the cell donor and the biochemical microenvironment are the major determinants of both bovine chondrocyte and MSC functional capacity [90].

### Diabetes mellitus

Currently, experimental and clinical data have provided support for the use of MSC for the treatment of diabetes mellitus [92]. Diabetes mellitus occurs in cattle and is similar to juvenile onset diabetes mellitus in humans, in that it is often immunomediated [93]. In cattle, no genetic background for diabetes has yet been confirmed [94]. Other etiologic factors have been implicated. Cases of diabetes have been reported in cattle infected with bovine diarrhea virus [94–96], and with foot and mouth disease [97]. Two mechanisms have been proposed to explain how the virus causes diabetes mellitus: (1) the beta cells in the pancreas are directly destroyed by the virus or (2) the immune response against the virus infection could induce an autoimmune response in the host [96]. The lack of insulin in animals with diabetes mellitus results in elevated glucose levels in the blood and urine. In addition, fatty acid synthesis in the liver is impaired in the diabetic animal and this leads to acid-base balance impairment, ketoacidosis, and dehydration, resulting in collapse, coma, and death [98]. MSCs were shown to transdifferentiate into islet-like clusters expressing insulin and glucagon [24]. At present, there are no clinical data available to validate MSC treatment for diabetes in bovine species. However, recent and promising evidence demonstrates that bovine MSCs have been successfully differentiated into islet-cells [20, 22]. More studies need to be done in order to prove the functionality of those cells for eventual use in preclinical trials and pharmaceutical studies.

## Potential of the bovine model for improvements to human health

The use of domestic animals as models has an essential role in narrowing the gap between translational research and clinical practice [99]. In regenerative medicine, the greatest advantage of using these models is to answer questions regarding the benefits and potential risks of stem cell treatments [100]. Each treatment needs to be tested in animal models, outlining human phenotypes, such as the size of the organs and more similar physiology [100]. Once the safety and efficacy of the treatment are proven, it can be applied in human therapy [100, 101]. The traditional model used for stem cell biology is the mouse, mainly because of its low cost, rapid reproduction, and ease of genetic modification [100]. Despite these advantages, the mouse model fails to precisely reproduce certain human diseases [100, 102]. Additionally, mice have a short lifespan, small body size, and different physiology when compared to humans [102]. Moreover, it is difficult to mimic the complexity of genetically heterogeneous human populations when studies are done with small groups of inbred mice [103]. To effectively study regenerative medicine and make the jump from the laboratory to human health applications, different animal models need to be used, allowing for better and more complete evaluations of cell-based therapies. In order to achieve this goal, it is important to select the most appropriate animal model, considering both size and experimental tractability, for example, ease of surgical manipulation, abundance of blood and tissues, efficiency of cloning, and feasibility of xenotransplantation [99]. Generally, larger animals are a better choice of model than mice for this purpose, specifically because they have a longer life span, which enables longitudinal studies, and because their physiological parameters are closer to those of humans [100]. Moreover, large animal species are more appropriate for mimicking human clinical settings due to their anatomy and physiology [99, 104].

The increase of genetic information can lead to new and more effective methodologies for the elimination or treatment of factors that negatively impact human health, such as cancer, cardiovascular disease, low birth weight, and infertility [99]. An important advantage of using cattle as a model is the possibility to study genetic and environmental influences on animal production and human disease [105]. The cattle genome contains a minimum of 22,000 genes, of which approximately 80% are shared with humans [106]. Due to these advantages over the mouse model, it is clear that more widespread adoption of the bovine model would have positive consequences for human health. In the field of tissue engineering, large animal models represent a promising tool that allows for the translation of novel experimental scaffolds into clinical practice [107].

An important advantage of large animals in tissue engineering is the fact that they provide large amounts of tissue that, after decellularization, can be used as scaffolds with similar organ size to that of humans as proven, for example, with the bovine placenta [66]. In order to elucidate physiological processes important to human health, the bovine model can be used for the study of reproduction regarding aging, physiology, gametogenesis, and infertility, as well as for bone structure formation, fat deposition, altitude and heat tolerance, hematopoiesis, leukemia, tuberculosis, xenotransplantation, gene therapy, and stem cells [99].

Although the use of a large animal model confers considerable advantages for translational applications, there are also some drawbacks that are important to consider when making a choice of model for an experiment. The major disadvantages of bovine models include the expenses of animal care, facility maintenance, necessity of veterinary support, and lesser availability of antibodies, probes, and reagents. However, due to the fact that they are more appropriate to mimic human scenarios than rodent models, these studies are essential to justify the risks and costs of clinical trials [108]. Research done in less translatable models such as mice necessitates repetition in more applicable organisms, leading to additional costs and delays developing critically needed therapies.

One example is stroke, which affects more than 795,000 people every year in the USA, costing $34 billion each year, frequently leaving victims permanently and severely disabled [109]. Current drug therapies are unable to regenerate lost tissue functionality, merely ameliorating the symptoms of the disease. Over the past 20 years, a number of promising studies have been published demonstrating the potential of MSC therapy to achieve recovery of the injured tissue, as reviewed in 2016 [110]. However, the vast majority of these studies have not been in translatable models, leading to a lack of progress towards new human therapies. With this in mind, future studies should focus on large animal models in order to evaluate the responses and safety of MSC therapy and advance the progress towards translational results. The ability to regenerate the damaged tissue suggests superior results to traditional therapies, and likely at a lower cost. The profound improvement in patient outcomes suggested by a potential switch to regenerative therapies for stroke victims provides just a single advantageous example of the many diseases in which cell therapies would vastly improve standards of care. This improvement would also, importantly, be accompanied by a significant reduction in the cost of treatment. On average, a stem cell treatment costs $5000 [111], so to treat 795,000 people per year would cost approximately $4 billion per year, resulting in massive savings in healthcare spending when compared to current therapies. Also noteworthy here is that cell therapies are still in their nascency and will likely continue to become less expensive as protocols are more completely developed and refined.

One potential area for cost reduction is an improved culture and transplantation methods. For example, the recently developed capacity to select a homogenous population of MSC without the necessity of cell sorting, accomplished through the selection of only the most adherent cells, can reduce the cost of cell production, not only because there is no need for expensive equipment and antibodies but also because in the first passage, a population is already selected, thereby reducing the cost of cell culture [19]. Additionally, it is known that bovine cells, when cultured at a higher density, can lead to less time and cost before transplantation [112].

It has been suggested that the National Institutes of Health could provide a national consortium of core laboratories with large animal models, facilitating the scientific community’s use of the models and furthering efforts to develop cell therapy and translation into human therapies [108]. This would provide a great improvement over the current, over-the-counter system, in which individual researchers are required to connect with individual livestock owners to arrange experiments. The opportunity to use cattle for regenerative medicine purposes may increase the efficiency of human therapy and reduce costs and work around the ethical issues of human clinical trials. Additionally, cell therapy in cattle creates the opportunity for producers to improve their production by applying cell-based therapy to their own animals, as previously discussed.

As discussed in this review, bovine mesenchymal stem cells have the potential to be differentiated into all three germ layers and can contribute to a large amount of studies in different areas of medicine that can be implemented in translational medicine, including bone and joint injuries, immunomediated diseases, type 1 diabetes, musculoskeletal disorders, infertility, and mastitis. Regenerative medicine and translational research need to interact in order to achieve an interdisciplinary perspective, investigating new insights into traditional clinical therapy and benefiting human and animal health.

## Conclusion

The fact that stem cell technology has developed significantly in non-bovine species creates both interest and background knowledge for the advancement of similar techniques in livestock. Mesenchymal stem cells are considered a promising source of cells for regenerative medicine. Initial interest in MSC was sparked decades ago due to both their inherent ethical appeal versus ESC and their suitability for laboratory work, resulting from the rapid cell culture and expansion that can take place after enzymatic disaggregation of tissue. This initial interest was compounded by revelations of diverse and medically relevant physiological effects such as their ability to proliferate in situ, modulate immune responses, and promote angiogenesis. Their potential clinical applicability and the scientific effort subsequently directed towards them were later expanded greatly when experiments proved that MSC could differentiate into cell types from all three germ layers, a typical minimum criterion for a cell type to be considered pluripotent. A potential reclassification of MSC as pluripotent is supported by results observed in bovine studies, which demonstrated again the ability of MSC to differentiate into all three germ layers and also showed them to display a gene profile consistent with pluripotency. The use of the bovine model for translational medicine has been shown to be advantageous, especially due to its abundance of biological material and similar size, anatomy, and physiology when compared to the traditional model. Isolation of bovine MSC has been performed from different tissues; however, the cells seem to express different markers according to the isolated tissue. More studies are needed to clarify species-specific protocols for bovine applications, in particular, because of the lack of availability of specific commercial antibodies. Additionally, their differentiation potential and clinical response need to be further investigated. It is clear from the information presented in the preceding articles that the ongoing development of bovine cell therapy shows promise for both veterinary clinicians and the livestock industry, especially for conditions that can result in loss of production from animals, such as mastitis and musculoskeletal disorders. The use of mesenchymal stem cells is an important tool both in the treatment of degenerative diseases and the improvement of functional recovery from traumatic injury. In addition, MSCs have the potential to be used to manipulate productivity in the cattle industry and to be used in nuclear transfer and also represent a tool for the preservation of valuable genetic resources. The lack of published studies and available clinical data in cows indicates both a deficiency and an opportunity of economic interest in this field of research. The next step will be to apply bovine MSC in clinical trials and evaluate the response of the animals as well as the economic impact of the techniques.

## Abbreviations

ASCs: Adult (somatic) stem cells
AT: Adipose tissue
AT-MSC: Adipose tissue-derived MSC
BM-MSC: Bone marrow-derived MSC
CFU-F: Fibroblastic colony-forming units
CO_2_: Carbon dioxide
ECM: Extracellular matrices
ESC: Embryonic stem cells
FBS: Fetal bovine serum
iPS: Induced pluripotent stem cells
*lfcinb*: Lactoferricin
MSC: Mesenchymal stem cell
NSP-MSC: Nasal septum-derived MSC
OA: Chronic osteoarthritis
TGF-β1: Transforming growth factor β
UC-MSC: Umbilical cord-derived MSC
